# Reviewer Acknowledgement 2013

**DOI:** 10.1186/1476-7120-12-4

**Published:** 2014-02-03

**Authors:** Rosa Sicari, Eugenio Picano

**Affiliations:** 1 Consiglio Nazionale delle Ricerche, Pisa, Italy

## Abstract

**Contributing reviewers:**

The Editors of *Cardiovascular Ultrasound* would like to thank all our reviewers who have contributed to the journal in Volume 11 (2013).

## 

Eustachio Agricola

Italy

Luigi Badano

Italy

Jacques Barth

United States of America

Elisabetta Bianchini

Italy

Adrian Borges

Germany

Alberto Bouzas-Mosquera

Spain

Rosa Maria Bruno

Italy

Stefan Buchner

Germany

Matteo Cameli

Italy

K. M. John Chan

United Kingdom

Nen-Chung Chang

Taiwan

Jiusheng Chen

United States of America

Quirino Ciampi

Italy

Lauro Cortigiani

Italy

Bernard Cosyns

Belgium

Koert de Waal

Australia

Serena Del Turco

Italy

Victoria Delgado

Netherlands

Vitantonio Di Bello

Italy

Erwan Donal

France

Andrejs Erglis

Latvia

Pompilio Faggiano

Italy

Francesco Faita

Italy

Silvia Favilli

Italy

Maurizio Galderisi

Italy

Helena Gardiner

United Kingdom

Luna Gargani

Italy

Stefano Ghio

Italy

Dipti Gupta

United States of America

Stephen Huang

Australia

Adrian Ionescu

United Kingdom

Jaroslaw Kasprzak

Poland

Hyung-Kwan Kim

South Korea

Aparna Kulkarni

United States of America

Claudia Kusmic

Italy

Patrizio Lancellotti

Belgium

Sangchol Lee

South Korea

Per Lindqvist

Sweden

Piotr Lipiec

Poland

Julien Magne

Belgium

Tom Matula

United States of America

Sergio Mondillo

Italy

Arnold Ng

Australia

Stefano Nistri

Italy

Vuyisile Nkomo

United States of America

Gianni Pedrizzetti

Italy

Mauro Pepi

Italy

Alessandro Pingitore

Italy

Jan Poelaert

Belgium

Bogdan Alexandru Popescu

Romania

Ulrica Prahl

Sweden

Lorenza Pratali

Italy

Min Qu

United States of America

Leonardo Roever

Brazil

David Russell

United Kingdom

Carlos Santos-Gallego

United States of America

Antti Saraste

Finland

Rosa Sicari

Italy

Ivan Stankovic

Serbia

Francesco Stea

Italy

Hidekazu Tanaka

Japan

Kenichi Tsujita

Japan

Stig Urheim

Norway

Albert Varga

Hungary

Yi-Xiang Wang

Hong Kong

Gillian Whalley

New Zealand

Meihua Zhu

United States of America

